# Development of Antiviral CVC (Chief Value Cotton) Fabric

**DOI:** 10.3390/polym13162601

**Published:** 2021-08-05

**Authors:** Wen-Yi Wang, Sui-Lung Yim, Chun-Ho Wong, Chi-Wai Kan

**Affiliations:** 1 Institute of Textiles and Clothing, The Hong Kong Polytechnic University, Hung Hom, Kowloon, Hong Kong, China; tcwang@polyu.edu.hk; 2 Avalon SteriTech Limited, Shatin, New Territories, Hong Kong, China; rogeryim@avalonbiomedical.com (S.-L.Y.); chwong@avalonbiomedical.com (C.-H.W.)

**Keywords:** PHMB, coronavirus, protective clothing, CVC fabric, antibacterial agent

## Abstract

The outbreak of COVID-19 has already generated a huge societal, economic and political losses worldwide. The present study aims to investigate the antiviral activity of Poly(hexamethylene biguanide) hydrochloride (PHMB) treated fabric against COVID-19 by using the surrogate Feline coronavirus. The antiviral analysis indicated that up to 94% of coronavirus was killed after contacting the CVC fabric treated with PHMB for 2 h, which suggests that PHMB treated fabric could be used for developing protective clothing and beddings with antiviral activity against coronavirus and can play a role in fighting the transmission of COVID-19 in the high-risk places.

## 1. Introduction

The global spread of COVID-19 has already resulted in serious societal, economic and political aftermath worldwide [1]. As of 9 April 2021, the confirmed cases of COVID-19 have exceeded 132 million globally and the official death toll now stands at nearly 3 million and will continue to grow [2]. Although a number of COVID-19 vaccines have been listed by the World Health Organization (WHO) for emergency use and many countries are starting to vaccinate, it is still necessary to perform personal protective measures, particularly in the high-risk public places. Textiles are a hotbed for the transmission of infectious diseases caused by microorganisms and viruses. Hence, there is a great need to develop antiviral protective clothing against COVID-19.

CVC (Chief Value Cotton) fabric is a blend of cotton and polyester with cotton equal to or over 50% of the blend. The high ratio of cotton to polyester threads imparts CVC fabric with many excellent performances such as breathability and durability. Moreover, the presence of polyester renders CVC fabric resistant to shrinkage and can withstand frequent household laundering. Due to these qualities, CVC fabric has become a staple in the sportswear industry and has been widely used for professional uniforms such as surgical gowns and medical scrubs. Cotton is capable of absorbing and retaining moisture and, thus, promotes the growth of microorganisms, which may result in some undesirable effects such as unpleasant odour, reduction in mechanical strength, stains and discolouration [3,4,5]. Moreover, the growth of microorganism can accelerate the hydrolysis of cellulose and cause deterioration of the fabric. Therefore, antibacterial finishing is of high importance for cotton and cotton blend fabric by eliminating the microbial colonization and the potential transfer from fabric surfaces.

Antibacterial finishing not only considers the selection of antibacterial agents but also emphasizes the durability of treated final products against wearing and household laundering. The ideal antibacterial agents should possess the virtues such as broad-spectrum germicidal efficacy, reasonable cost, biocompatibility and low toxicity. The affinity of selected antibacterial agents to fibers plays a vital role in the durability of antibacterial fabrics. In this regard, the hydrophilic polymeric antibacterial agents provide a better choice than the inorganic metal nanoparticles [6,7]. A wide variety of polymeric antibacterial agents have been developed, ranging from naturally derived biopolymers, such as chitosan and starch and synthetic polymers including quaternary ammonium salts, polybiguanide, triclosan and N-halamine, to inorganic metal nanoparticles, such as TiO_2_, ZnO and silver nanoparticles [6,7]. It has been reported that some antibacterial agents, e.g., triclosan and copper-based compounds, have been also used for the development of antiviral fabrics due to their antiviral activities [8]. Lyigundogdu and colleagues reported a triclosan-based antiviral formulation containing 0.03% triclosan, 3% sodium pentaborate pentahydrate and 7% glucapon for developing antiviral cotton fabrics [9]. The antiviral fabrics shows excellent results against adenovirus and poliovirus.

Poly(hexamethylene biguanide) hydrochloride (PHMB) is a commercial polycationic biocide because of its broad-spectrum biocidal activity and reasonable cost, which has been widely used in food industry, swimming pool sanitizers, leather preservatives, contact lens disinfectants, wound dressings and textile industry as an antibacterial agent [10,11]. PHMB consists of repeating biguanidine units separated by hexamethylene hydrocarbon chains. The antimicrobial activity of PHMB is highly associated with the cationic biguanide segments in the molecular structure, which can interact with the negatively charged phosphate head of bacterial phospholipids cell membrane and finally cause cell death [12,13]. The application of PHMB in textile finishing can be achieved by the conventional exhaust method of padding and spraying or pad-dry-cure process [14,15]. The adsorption of PHMB on cellulosic surface consists of a combination of typical Langmuir isotherms and Freundlich isotherms in a concentration-dependent manner, attributed to formation of monolayer aggregation and multilayer stacking of PHMB by electrostatic interaction and hydrogen bonding with a close 1:1 stoichiometry between charges [16,17,18]. This accounts for its strong affinity to cellulosic fibers and good durability against abrasion and household laundering after fabrics coating [19,20]. Therefore, it has been reported to develop wash-durable antimicrobial fabrics coated with PHMB [21]. Additionally, PHMB was shown to be active against HIV-1, and other biguanide derivatives were found to be effective against vaccinia and influenza viruses [22]. Pinto studied the virucidal properties and the mechanism of action of PHMB-based biocides in detail and found that PHMB shows modest virucidal activity against enveloped human virus by interacting with viral capsid and resulting in virus death [23]. Considering the good biocidal efficacy and biosafety of PHMB, we are hereby inspired to investigate the applicability of PHMB used for developing an antiviral fabric against COVID-19.

## 2. Materials and Methods

CVC woven fabric with weight 272.8 g/cm^2^ (cotton 50%/polyester 50%) was used in this study. The impurities on the fabric were removed by 5 g/L of sodium hydroxide solution (chemical grade and obtained from Sigma-Aldrich, St. Louis, MO, USA) at 60 °C for 30 min, and then the fabric was thoroughly rinsed with tap water and dried at room temperature. Antibacterial agent PHMB (20% w/v aqueous solution), polyethylene glycol 400 (PEG400) (400 g/mol in average), polyurethane binder (20% w/v aqueous solution) and bromophenol blue (BPB) sodium were obtained from Sigma-Aldrich (St. Louis, MO, USA).

### 2.1. Coating Treatment

The finishing formulation was prepared with 10% (w/v) PHMB, 5% (w/v) PEG400 and 8% (w/v) binder and used to treat the fabric samples by “pad-dry-cure” method. Specifically, the fabric sample was padded in a laboratory-scale horizontal padder with 80% wet pickup and then dried in a laboratory oven at 90 °C for 5 min and cured at 130 °C for 45 s. After antibacterial coating, the fabric samples were stored under standard conditions at a temperature of 20 ± 2 °C and 65% ± 2% relative humidity for at least 24 h prior to subsequent testing.

### 2.2. Qualitative Determination of PHMB

The presence of PHMB on the fabric was qualitatively determined by the anionic dye BPB, which can be complexed with PHMB with the formation of a blue stable complex. The present study utilizes BPB to qualitatively determine the presence of PHMB coated on the fabrics.

### 2.3. Antiviral Activity Measurement

The antiviral activity of PHMB-coated fabric samples was conducted against Feline coronavirus by Microbiological Solutions Ltd. (MSL, Lancashire, UK) according to ISO 18184:2019. The antiviral fabrics (20 × 20 mm) were inoculated with 200 μL of virus at a concentration of 10^5^ and left for 2 h at 25 °C. After contacting for 2 h, 20 mL of wash-out solution in the vial containers was added with agitation by Vortex mixer for 5 s and 5 times to wash out the virus from the specimens. TCID_50_ was calculated following the appropriate incubation time. The antiviral activity value was then calculated by comparison of the antiviral test recovered from the control fabric according to Equation (1):

*M* = lg(*V_b_*) − lg(*V_c_*), (1)

where *M* is antiviral activity value; lg(*V_b_*) is the common logarithm average of three infectivity titer after contacting with the reference specimen (PFU/vial) for 2 h; and lg(*V_c_*) is the common logarithm average of three infectivity titer contacting with the antiviral fabric specimen for 2 h.

The antiviral activity percentage was determined according to Equation (2):

(2) $$\mathrm{Percentage}\%\;=\;\frac{V_{b}\;-\;V_{c}}{V_{b}}\;\times 100\%$$

where *V_b_* (PFU/vial) is the average of three infectivity titer after contacting with the reference specimen for 2 h, and *V_c_* (PFU/vial) is the average of three infectivity titer contacting with the antiviral fabric specimen for 2 h.

## 3. Results and Discussion

**Figure 1:**
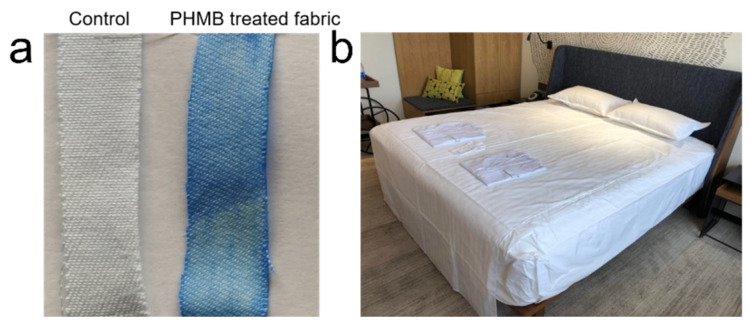
Images of the (**a**) untreated fabric and PHMB treated fabric sample after complexing with BPB and (**b**) PHMB treated CVC fabric on hotel bedsheet linen.

PHMB treated CVC fabrics are shown in Figure 1. Clearly, compared to the control sample, PHMB treated fabric shows a distinct blue shade, indicating that PHMB was successfully coated on the surface of fabric sample (Figure 1a). Afterwards, the antiviral activity of PHMB treated fabrics against coronavirus was investigated by using Feline coronavirus as a surrogate of SARS-CoV-2. Table 1 shows the antiviral activity values of the control and PHMB treated fabric samples. The reduction value for the control sample before and after contacting was 0.51, which is satisfactory and ensures the validity of the testing. By contrast, the result for the fabric sample treated with PHMB showed an overall 1.22 log reduction against Feline coronavirus with a 2 h contact time, which corresponds to 94.01% of reduction percentage. This demonstrates that the fabrics treated with PHMB could effectively disable the virus and help contain the transmission of coronavirus.

It has been reported that COVID-19 could survive for 48 h on cloths [24]. The present study shows that PHMB treated fabric can inactivate 94% of feline coronavirus after contacting for 2 h. Based on this study, PHMB coating can enhance the added values of CVC fabrics with antibacterial and antiviral properties. This technology has been transferred and applied to antiviral CVC fabrics used in hotel and dormitory linen, as shown in Figure 1b. Given that the susceptibility of COVID-19 relative to standard disinfection methods [24], it is believed that PHMB treated CVC fabric could play a role in fighting the transmission of COVID-19, particularly in the high-risk places such as hospital and hotels.

## 4. Conclusions

The present study investigates the antiviral activity of PHMB treated fabric against Feline coronavirus. The presence of PHMB coated on the surface of fabric was qualitatively determined by BPB. The antiviral test indicated that the CVC fabric treated with PHMB showed a 94% reduction in feline coronavirus upon 2 h contact. This evidenced that PHMB treated fabric could be used for developing protective clothing and beddings with antiviral activity against coronavirus.

## Figures and Tables

**Table 1:** Antiviral activity values of the control and PHMB treated fabric samples.

Sample	Logarithm Average of Infectivity Titer (PFU/Vial)		Antiviral Activity	Percentage (%)
Control	Immediately after inoculation	5.29	N/A	N/A
	After contacting for 2 h	4.78	0.51	69.37
PHMB treated fabric	After contacting for 2 h	4.07	1.22	94.01
